# Inter- and Intra-Annual Bacterioplankton Community Patterns in a Deepwater Sub-Arctic Region: Persistent High Background Abundance of Putative Oil Degraders

**DOI:** 10.1128/mBio.03701-20

**Published:** 2021-03-16

**Authors:** Angelina G. Angelova, Barbara Berx, Eileen Bresnan, Samantha B. Joye, Andrew Free, Tony Gutierrez

**Affiliations:** a School of Engineering and Physical Sciences, Heriot-Watt University, Edinburgh, United Kingdom; b Marine Scotland Science, Aberdeen, United Kingdom; c Department of Marine Sciences, University of Georgia, Athens, Georgia, USA; d School of Biological Sciences, University of Edinburgh, Edinburgh, United Kingdom; Oregon State University

## Abstract

Hydrocarbon-degrading bacteria naturally degrade and remove petroleum pollutants, yet baselines do not currently exist for these critical microorganisms in many regions where the oil and gas industry is active. Furthermore, understanding how a baseline community changes across the seasons and its potential to respond to an oil spill event are prerequisites for predicting their response to elevated hydrocarbon exposures. In this study, 16S rRNA gene-based profiling was used to assess the spatiotemporal variability of baseline bacterioplankton community composition in the Faroe-Shetland Channel (FSC), a deepwater sub-Arctic region where the oil and gas industry has been active for the last 40 years. Over a period of 2 years, we captured the diversity of the bacterioplankton community within distinct water masses (defined by their temperature and salinity) that have a distinct geographic origin (Atlantic or Nordic), depth, and direction of flow. We demonstrate that bacterioplankton communities were significantly different across water samples of contrasting origin and depth. Taxa of known hydrocarbon-degrading bacteria were observed at higher-than-anticipated abundances in water masses originating in the Nordic Seas, suggesting these organisms are sustained by an unconfirmed source of oil input in that region. In the event of an oil spill, our results suggest that the response of these organisms is severely hindered by the low temperatures and nutrient levels that are typical for the FSC.

## Abstract (precis)

Oil spills at sea are one of the most disastrous anthropogenic pollution events, with the Deepwater Horizon spill providing a testament to how profoundly the health of marine ecosystems and the livelihood of its coastal inhabitants can be severely impacted by spilled oil. The fate of oil in the environment is largely dictated by the presence and activities of natural communities of oil-degrading bacteria.

## INTRODUCTION

The Faroe-Shetland Channel (FSC), which lies to the west of Shetland, has a significant history of oil exploration and production over the last 40 years (1, 2). With oil exploration forecast to expand into deeper waters of the FSC, the risk of oil spills in this region is likely to increase. Hydrocarbon-degrading bacteria play a major role in the remediation of hydrocarbon pollution in the ocean (3–6). Despite a recent burst in knowledge and understanding of the diversity, behavior, and function of marine microbial communities in response to hydrocarbon contamination in marine environments (4, 7–9), regions like the Arctic, sub-Arctic, and deep ocean basins remain largely underexplored in this respect, due mainly to the challenge of sampling and unpredictability associated with these environments. Past major oil spills (e.g., *Exxon Valdez* and Deepwater Horizon) highlighted the need for a prerequisite understanding of the structure, diversity, and dynamics of prespill baseline bacterial communities (4, 10, 11).

**FIG 1:**
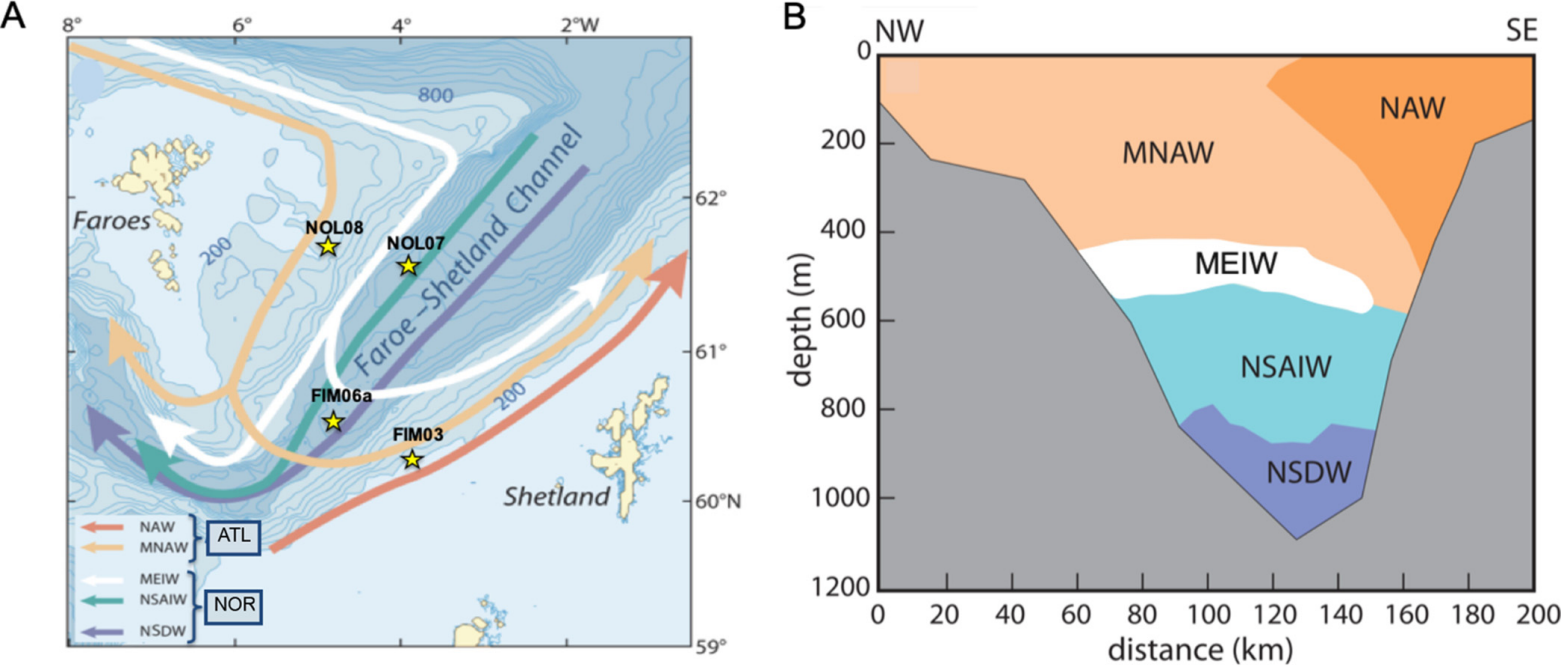
(A) Map of the Faroe Shetland Channel showing aerial view, including bathymetry lines, a schematic representation of water mass direction of flow, and sampling stations. Sampling locations for microbial baseline profiling are marked by yellow stars. (B) Cross-sectional view of water mass layers and relative depths in the water column of the FSC, as adapted from Berx (12); data underlying this figure are from Hughes et al. (78). Abbreviations: NAW, North Atlantic Water; MNAW, Modified North Atlantic Water; MEIW, Modified East Icelandic Water; NSAIW, Norwegian Sea Arctic Intermediate Water; NSDW, Norwegian Sea Deep Water. The images in this figure are subject to Crown copyright.

Despite extensive century-long hydrographic monitoring of the FSC (12–14) and considering the potential risk for oil contamination into its deep waters, a basic understanding of this region’s microbial composition and dynamics remains lacking. The FSC is a region of complex hydrography: five water masses, of defined temperature and salinity properties and originating from different source regions, flow through and mix within the channel (Fig. 1). The five water masses can be grouped into two broader categories by their source region: the North Atlantic Ocean or the Nordic Seas (NOR; Greenland, Iceland, and Norwegian Seas). The North Atlantic Water (NAW) and the Modified North Atlantic Water (MNAW) both originate from the Atlantic Ocean (ATL) but can be found at different depths or directions of flow throughout the FSC, depending on location. Although their properties, depth, and path through the FSC are relatively distinct, they can be identified more broadly as warm, saline waters flowing close to the surface between 0 and 400 m (Table 1).

**TABLE 1:** Geographical coordinates, depth, and station names of sampling locations for microbial profiling of each targeted FSC water mass^*a*^

Station name	Sampling depth (m)	Targeted (range)			Observed (range)		Geographical coordinates
		Water mass	Temp (°C)	Salinity	Temp (°C)	Salinity	
FIM03	150	NAW	>9.5	35.35–35.45	8.7–10.0	35.36–35.39	60° 20.25' N, 04° 09.00' W
NOL08	175	MNAW	7.0–8.5	35.1–35.3	7.5–8.7	35.2–35.3	61° 42.00' N, 04° 51.00' W
NOL07	700	NSAIW	−0.5–0.5	34.87–34.90	−0.5–0.0	34.90–34.91	61° 35.00' N, 04° 15.00' W
FIM6a	1,100	NSDW	<−0.5	34.91	<−0.5	34.91	61° 38.00' N, 04° 54.00' W

a Targeted and observed temperature and salinity characteristics during research cruises in 2014 and 2015 are included. Abbreviations: NAW, North Atlantic Water; MNAW, Modified North Atlantic Water; NSAIW, Norwegian Sea Arctic Intermediate Water; NSDW, Norwegian Sea Deep Water.

The deepest water masses, the Norwegian Sea Arctic Intermediate water (NSAIW) and the Norwegian Sea Deep Water (NSDW), originate from the Nordic Seas. These are found deeper than ∼600 m down to the ocean floor at ∼1,200-m depth. These water masses are characterized by their specific depth (∼600 to ∼850 m for NSAIW and ∼850 to 1,200 m for NSDW) and more broadly by their low temperature and salinity. The final water mass, Modified East Icelandic Water (MEIW), between depths of ∼400 m and ∼600 m (15, 16), is affected by seasonal and annual fluctuations in glacier and polar water inflow. The dynamic mixing of MEIW with the contrasting Atlantic- and Nordic-origin water masses produces fluctuations in its volumetric presence and composition, making it the most compositionally and/or temporally variable water mass in the region (12, 15–17).

While numerous studies have described the microbial community patterns in shallow waters (18–22), a similar level of understanding is a major knowledge gap for deep, hydrodynamically complex systems such as the FSC. Pioneering studies exploring the microbiomes of large oceanic systems (e.g., Tara Oceans Project and the Earth Microbiome Project) (19, 23–27) have generated an unprecedented view of depth-dependent, temporal, and geographic diversity and variation in microbial populations. These international and collaborative efforts and the International Census of Marine Microbes (23, 28) provided a very comprehensive picture of baseline bacterioplankton communities and their metabolic functions and associations in the global ocean ecosystem. Such studies have reported patterns of microbial community composition and functionality, driven primarily by temperature rather than geographic or other environmental factors (29).

Due to the large-scale nature of such studies, however, probing deeper into the diversity and the effects of different environmental factors on specific taxa has understandably left a lot of regions underrepresented. Elucidating the functional composition of the microbiome in more regions, especially those prone to major anthropogenic threats and perturbations, is important in the present-day changing ocean. For example, the Deepwater Horizon disaster brought to light the lack of a baseline understanding of microbial distributions in the Gulf of Mexico and the limitations in our ability to predict how microbial communities would respond to perturbations and influence various Gulf ecosystems and economies (4, 9, 11, 30). The work also illustrated the importance of obtaining region-specific baseline data for microbial community composition, stratification, and temporal dynamics in high-risk oil pollution regions. This information is valuable not only for improving the development of enhanced oil spill remediation and mitigation strategies but also for assessing ecosystem recovery (11).

In 2011, Agogué et al. explored microbial communities of the North Atlantic Ocean, including from a few sub-Arctic regions, with water flowing toward and potentially through the FSC (25). The authors reported bio-oceanographic islands of microbial communities, defined by the water masses present within the deep water of the Atlantic Ocean. Due to the proximity and flow direction of the region, in the present study we used these observations to design a water mass-based monitoring strategy to conduct a 16S rRNA gene-based survey of the microbial communities and their pattern of variability in the FSC over a period of 2 years. This is the first microbial survey of the FSC covering a longitudinal and vertical water column transect for the region. We identify recognized and putative hydrocarbon-degrading bacteria in the different water masses across seasons and explore their compositional patterns to assess their potential to respond to oil spills in this vulnerable sub-Arctic region.

## RESULTS

### Water mass physical data.

Statistical analysis (Student’s *t* test) revealed no significant differences (all *P* values of >0.05) between the expected water characteristics (salinity and temperature) for each targeted water mass and the observed value over the course of the 2-year monitoring period. An exception was the observed salinity characteristics for the NSAIW mass (Table 1), which consistently showed significant deviation (*P* < 0.05) from the expected salinity values, presumably due to the mixing of exogenous water within the NSAIW. The measured temperature and depth characteristics, however, strongly identified the targeted water as NSAIW (Table 1). Based on these characteristics, the microbial communities in the sampled waters were accepted as the best representation of their reciprocal targeted water mass. As a result, a total of 48 water samples (representing triplicates) were obtained from the FSC during research cruises in 2014 and 2015, intended to produce the first spring and fall microbial community baseline for a northeast Atlantic region.

### Seasonal, hydrographic, and bacterioplankton community correlations.

MiSeq sequencing from 32 out of the 48 FSC samples (representing duplicates) returned a total of 11,037,306 individual sequencing reads. After preprocessing (merging, trimming, and short-read and quality filtering), a total of 4,739,293 good-quality merged sequences were obtained, representing ∼86% of all raw sequence data generated. After demultiplexing, denoising/clustering, and chimera filtration, the resultant good-quality V3-V4 16S rRNA gene fragment sequences produced 1,514 amplicon sequence variants (ASVs). After additional filtering (ASV prevalence at ≤20% and abundance at <15%), the number of ASVs obtained was 1,431.

**FIG 2:**
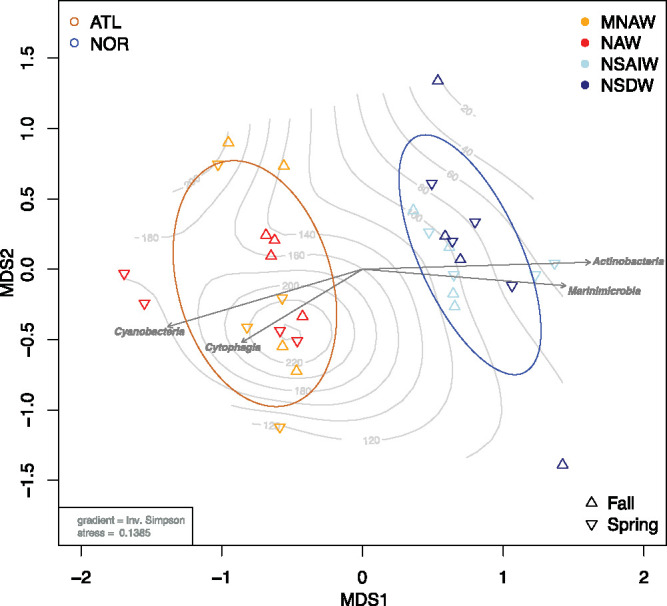
Nonparametric multidimensional scaling ordination based on Bray-Curtis dissimilarity matrix of FSC microbial communities from different water masses and seasons. Shapes represent seasons. Colors represent water masses. Arrows represent significant positive correlation between the indicated taxonomic rank and the corresponding community, determined by Mantel test. Abbreviations: NAW, North Atlantic Water; MNAW, Modified North Atlantic Water; NSAIW, Norwegian Sea Arctic Intermediate Water; NSDW, Norwegian Sea Deep Water; ATL, Atlantic origin; NOR, Nordic origin.

Permutational multivariate alpha diversity variance analysis (PERMANOVA) tests applied to filtered ASV abundance showed significant differences in communities based on water mass origin (*P = *0.001) or specific water masses (*P = *0.001). Pair-wise PERMANOVA tests applied between water mass origins showed that communities of water masses belonging to the same origin (ATL or NOR) were not statistically distinguishable from each other (*P = *0.47 for NAW versus MNAW, *P = *0.055 for MSAIW versus NSDW). Water masses derived from contrasting sources (e.g., MNAW versus NSAIW) contributed to the observed community differences within the FSC (*P < *0.05 in all cases). Notably, this was also highlighted in a nonmetric multidimensional scaling (nMDS) ordination plot (Fig. 2) in which the communities of water masses from similar origin cluster closer together, indicating a stronger community affiliation toward water origin than water mass or even water depth. This was especially true for the NOR communities, which differed more drastically in depth yet presented a tighter cluster. Hence, and unless otherwise specified in the text, downstream community analysis for the FSC was generalized in terms of water origin (ATL or NOR) rather than individual water masses.

Initial investigations for the presence of seasonal patterns within the communities of the FSC, performed on both the Bray-Curtis and UniFrac distance matrixes, indicated no season-based community variations (Bray-Curtis-based *P = *0.11; UniFrac-based omni-*P = *0.125). The microbial communities in ATL water, however, varied significantly between the fall and spring seasons (Bray-Curtis-based PERMANOVA *P = *0.042; UniFrac-based omni-*P = *0.001), with seasonal variations explaining as much as 14% of all compositional changes (analysis of similarity tests [ANOSIM] R value, 0.145; *P = *0.05). In contrast, the NOR communities showed no season-based community variations (Bray-Curtis-based PERMANOVA *P = *0.175, UniFrac-based omni-*P = *0.083), and season changes demonstrated only 6% contribution to community variations within the NOR water (ANOSIM R value, 0.057; *P = *0.184). When the water from both origins was considered together, the overall microbial community of the FSC presented no pattern of seasonality. More detail into the seasonal variations within the ATL communities is presented and discussed below.

### Bacterioplankton community profiles.

**FIG 3:**
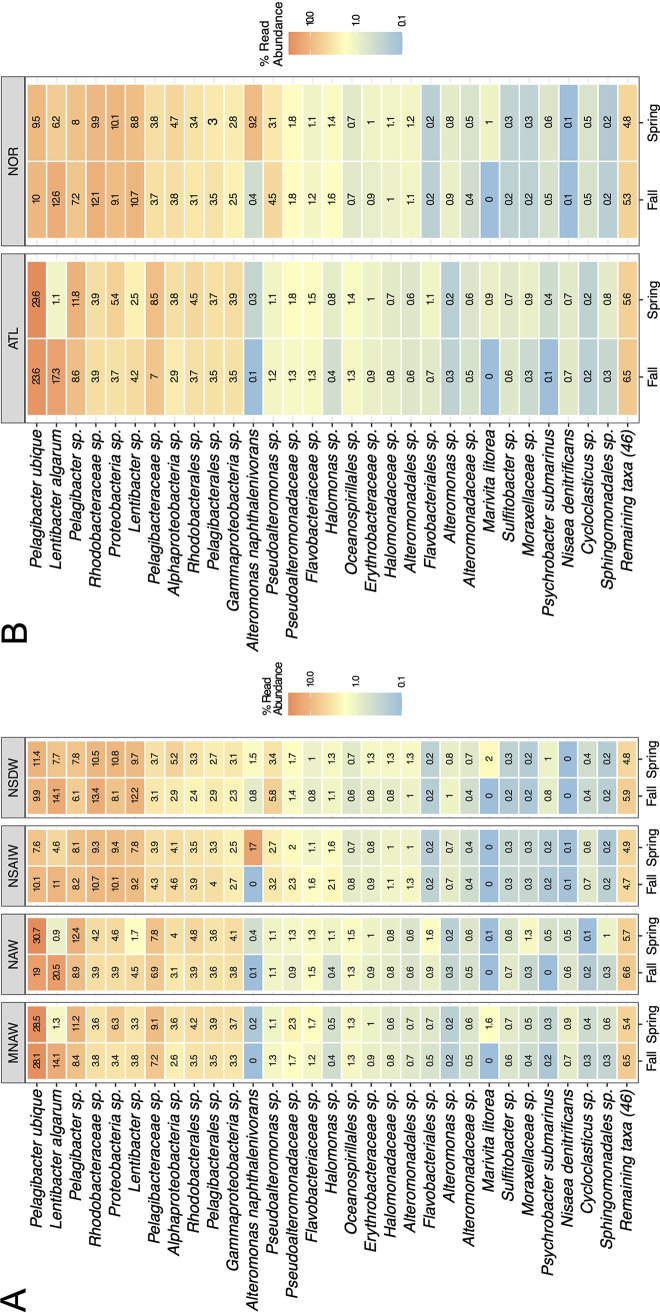
Heatmap representing the seasonal average of the microbial composition throughout the FSC water column (normalized to 100%) from 16S rRNA gene fragment-based sequencing, presented at the species level (or deepest possible taxonomic rank per ASV) with respect to water masses through the entire FSC (A) and water mass origin (source) in the entire FSC water column (B). Taxonomies beyond the top 30 abundance ranks are grouped under “Remaining taxa.” Abbreviations: NAW, North Atlantic Water; MNAW, Modified North Atlantic Water; NSAIW, Norwegian Sea Arctic Intermediate Water; NSDW, Norwegian Sea Deep Water; ATL, Atlantic origin; NOR, Nordic origin. Taxonomy of ASVs is shown to the most accurate level that could be assigned.

The microbial profiles for the sampled FSC waters are presented in Fig. 3A with respect to water masses and seasons and in Fig. 3B with respect to water mass origin and seasons. Apart from *Proteobacteria* (80% relative abundance), the dominant phyla in the ATL also included *Bacteroidetes* (∼4%), *Nitrospinae* (∼4%), and Archaeal Marine Group II (∼4%) (Fig. 3B). About 2% of the bacterial community within the ATL remained unclassified beyond the domain level. The most abundant taxonomic classes in the ATL were *Alpha*- and *Gammaproteobacteria* (54% and ∼21%, respectively). Dominant *Alphaproteobacteria* phylotypes were represented primarily by the clade SAR11 and the order *Rhodobacterales*, dominated by species *Pelagibacter ubique* (19 to 30% of the total microbial community) and *Lentibacter algarum* (1 to 20%), respectively. The most abundant *Gammaproteobacteria* were represented by members belonging to the orders *Oceanospirillales*, *Alteromonadales*, and *Pseudomonadales*, with unrecognized species of the genera *Pseudoalteromonas* and *Halomonas*. Of the *Bacteroidetes* phylum, *Flavobacteriia* were found to be prevalent (∼3% of total diversity).

The most abundant phylum in the NOR was *Proteobacteria*, contributing ∼78% of the total microbial community, followed by the phyla *Actinobacteria* (6%), *Bacteroidetes* (∼3%), and *Marinimicrobia* (∼2%). Archaeal Marine Group II and *Flavobacteriia* remained at constant abundances (3 to 4%) of the total microbial community in the NOR. The most dominant class in the NOR was the *Alphaproteobacteria*, although its relative abundance decreased (down to ∼32%) and almost equaled that of the *Gammaproteobacteria* (∼29%) compared to the ATL. The *Alphaproteobacteria* were dominated by members of the order *Rhodobacterales* (∼18%) and specifically by members of the genus *Lentibacter* (∼10% of total microbial community). The SAR11 clade, dominating at more than a quarter of the total community in the ATL, represented only ∼9% of the bacterioplankton community in the NOR. Of the *Gammaproteobacteria*, the most dominant phylotypes were Alteromonas naphthalenivorans (0 to 17%) and members of the genera *Pseudoalteromonas* (∼5%), *Alteromonas* (∼5%), and *Halomonas* (∼3%). About 6% of all *Gammaproteobacteria* remained unclassified at and beyond class level (Fig. 3). Of all the bacteria detected in the NOR, those that could not be classified beyond domain level were double in relative abundance compared to that in the ATL, accounting for ∼4% of the entire microbial community.

The alpha diversity of communities across the waters with different origins suggested similar species richness (ANOVA *P = *0.65) but with discrepancies in evenness values, resulting in significantly different diversity indices (both Shannon and inverted Simpson ANOVA *P = *0.001). Seasonal changes within the communities of each water mass origin were not apparent in any of the alpha diversity indices.

### Seasonal and spatially structured hydrocarbon degraders and other communities.

Seasonal variations in the microbial profiles between the spring and fall for each water mass origin are shown in Fig. 3. *Alphaproteobacteria* dominated the spring communities of the ATL (∼50%), with their dominance highest during the fall, at an average of 58% relative abundance. This apparent seasonally influenced change was due to a 13-fold increase in abundance of *Lentibacter*, from ∼1% during the spring to ∼17% in the fall (Fig. 3). SAR11 levels also varied slightly during the colder months within the ATL, changing between ∼31% for the spring and ∼26% in the fall. The abundance of the *Gammaproteobacteria* within the ATL was slightly varied across the seasons, with ∼24% during the spring decreasing to ∼18% in the fall. However, the internal composition and proportions of representative gammaproteobacterial subranks (*Thiomicrospirales*, *Alteromonadales*, and *Oceanospirillales*) remained consistent across these seasons. Archaeal Marine Group II was found in highest abundance during the fall within the ATL (∼2% in spring, increasing to ∼5% in fall). Seasonal variation within the NOR was observed most profoundly for the *Gammaproteobacteria*, decreasing in relative abundance from ∼41% in the spring to ∼23% in the fall. In contrast, *Alphaproteobacteria* increased in abundance in the fall within the NOR (from 36% in the spring to 55% in the fall), with the colder months bringing about primarily an increase in abundance of *Lentibacter* (from ∼5% to ∼21%) (Fig. 3).

Significant changes in community composition were also reflected in some of the rarer phylotypes (abundance, <1%), especially within the NOR. Differential abundance analysis (based on the DESeq2 algorithm) was applied to assess this variation (Table 2). Some of the most significant changes identified in the ATL over the spring were an increase in the abundance of members associated with *Alphaproteobacteria* (e.g., *Sphingomonadales* spp., Sulfitobacter dubius, and Marivita litorea) (Table 2). Of the *Gammaproteobacteria*, *Alcanivorax*, a genus comprising obligate hydrocarbon degraders (5), and *Psychrobacter* significantly increased during the spring in the ATL. During the fall, the ATL was represented by a significant increase in the gammaproteobacterial subgroups *Vibrio*, *Colwellia*, *Piscirickettsiaceae*, and *Pseudoalteromonos*, all of which contain multiple members with reported hydrocarbon-degrading abilities (31). In the NOR during the fall, a significant elevation in *Alphaproteobacteria* (e.g., *Novosphingobium* and *Amylibacter*) and members of the gammaproteobacterial hydrocarbonoclastic group *Colwellia* was detected (Table 2) (31). In spring in the NOR, the *Gammaproteobacteria* with known hydrocarbon-degrading qualities, such as Alteromonas naphthalenivorans, were also recognized as significantly elevated (31).

**TABLE 2:** Differential abundance analysis (DESeq2) results for the species with significant seasonal fluctuations from each water mass origin within the FSC^*a*^

Water origin and species	Adjusted *P* value	Season of upregulation
ATL		
*Colwellia* sp.	8.05E−06	Spring
Marivita litorea	1.39E−04	Spring
*Sphingomonadales* sp.	1.39E−04	Spring
*Proteobacteria* sp.	7.84E−03	Spring
Sulfitobacter dubius	8.86E−03	Spring
Alteromonas naphthalenivorans	1.35E−02	Spring
*Alphaproteobacteria* sp.	1.53E−02	Spring
Psychrobacter submarinus	2.22E−02	Spring
*Limimaricola hongkongensis*	2.39E−02	Spring
*Moraxellaceae* sp.	4.52E−02	Spring
Alcanivorax venustensis	4.52E−02	Spring
*Pelagibacter* sp.	4.52E−02	Spring
*Nisaea* sp.	4.75E−02	Spring
Vibrio splendidus	2.16E−14	Fall
*Lentibacter algarum*	2.11E−05	Fall
Colwellia psychrerythraea	8.86E−03	Fall
*Piscirickettsiaceae* sp.	1.35E−02	Fall
*Sphingomonas* sp.	1.97E−02	Fall
Pseudoalteromonas denitrificans	2.22E−02	Fall
NOR		
*Nitrospinaceae* sp.	1.80E−05	Spring
Alteromonas naphthalenivorans	2.84E−03	Spring
Novosphingobium capsulatum	7.20E−12	Fall
*Limimaricola* sp.	8.44E−04	Fall
Colwellia aquaemaris	8.44E−04	Fall
Sphingomonas oligophenolica	2.10E−02	Fall
Amylibacter ulvae	2.10E−02	Fall

a Abbreviations: ATL, Atlantic origin; NOR, Nordic origin.

Bacterial genera with known hydrocarbon-degrading capabilities were significantly more abundant (PERMANOVA *P = *0.003) in the NOR than the ATL (14.8% versus 4.6%, respectively) (Table 3). Significant variation in the abundance of hydrocarbonoclastic bacteria was also found across seasons within the ATL (*P = *0.001) but not within the NOR (*P* = 0.061) (Table 3). The level of the hydrocarbon-degrading community was higher during the spring in the ATL, decreasing from ∼5.1% in the spring to ∼4.3% in the fall, which, as discussed below, indicates psychrophilic behavior or seasonal nutrient availability. In the NOR, the hydrocarbon-degrading population was more stable across the two seasons, representing ∼15% of the total microbial community. The most abundant genera with members comprising reported hydrocarbon-degrading capabilities were *Alteromonas* and *Pseudoalteromonas*. Taken collectively, on average, 10% of the bacterial genera in the FSC contained putative and/or confirmed hydrocarbon-degrading abilities.

**TABLE 3:** Average relative abundance and taxonomic affiliation of most abundant (>0.01%) bacterial taxa, with reported hydrocarbon-degrading qualities identified in the water column of the FSC^*a*^

Taxonomic affiliation					FSC (*n* = 32)	AWC (*n* = 16)	NWC (*n* = 16)	AWC spring (*n* = 8)	AWC fall (*n* = 8)	NWC spring (*n* = 8)	NWC fall (*n* = 8)
Phylum	Class	Order	Genus	Reference							
*Actinobacteria*	*Actinobacteria*	*Actinomycetales* (2)		79, 80	0.2	0.0	0.3	1.0	1.0	3.0	3.0
*Bacteroidetes*	*Flavobacteriia*	*Flavobacteriales*	*Polaribacter* (14)	31, 80, 81	0.4	0.7	0.1	0.3	0.0	0.4	1.0
			*Tenacibaculum* (2)	31, 80	0.1	0.2	0.0	0.4	0.0	0.4	1.0
*Proteobacteria*	*Alphaproteobacteria*	*Rhodobacterales*	*Sulfitobacter* (6)	30, 31, 43, 81	0.5	0.3	0.6	0.6	0.2	0.6	0.7
			*Amylibacter* (13)	82	0.4	0.8	0.0	0.7	0.8	0.0	0.1
		*Sphingomonadales*	*Novosphingobium* (4)	5, 79	0.1	0.0	0.2	0.0	0.1	0.0	0.4
			*Sphingomonas* (3)	3, 5, 79, 80, 83	0.4	0.3	0.4	0.0	0.1	0.0	0.6
	*Betaproteobacteria*	*Nitrosomonadales* (3)		5, 79, 84	0.1	0.2	0.0	0.0	0.3	0.0	0.1
	*Gammaproteobacteria*	*Alteromonadales*	*Alteromonas* (27)	4, 6, 43, 79	2.6	0.1	5.0	0.3	0.0	2.0	0.4
			*Colwellia* (7)	4, 31, 80, 85	0.4	0.1	0.8	0.1	0.1	0.1	0.0
			*Marinobacter* (1)	3–5, 30, 31, 79, 80, 86	0.1	0.0	0.1	0.1	0.0	0.2	0.1
			*Pseudoalteromonas* (18)	5, 6, 80, 81	3.0	1.0	5.0	1.0	0.7	0.2	0.1
		*Pseudomonadales*	*Psychrobacter* (10)	80	0.7	0.3	1.0	0.5	0.4	0.4	0.4
		*Oceanospirillales*	*Alcanivorax* (6)	3–6, 31, 43, 79, 87	0.1	0.1	0.1	0.1	0.0	0.1	0.0
			*Halomonas* (27)	3–6, 79, 80, 88	0.5	0.0	1.0	0.0	0.0	0.7	1.0
			*Oleispira* (2)	3–5, 31, 79, 80	0.1	0.0	0.1	0.1	0.0	0.1	0.1
		*Thiotrichales*	*Cycloclasticus* (7)	3–6, 9, 43, 79, 85	0.0	0.0	0.0	0.0	0.0	7.0	5.0
		*Vibrionales*	*Vibrio* (2)	5, 30, 79, 81, 83	0.3	0.4	0.1	0.0	0.6	0.0	0.4
Total % hydrocarbonoclastic fractions					9.71	4.6	14.8	5.1	4.3	15.2	14.5

a Number of ASVs aggregated into the relative abundance numbers are presented in parenthesis next to genus name. Abbreviations: ATL, Atlantic origin; NOR, Nordic origin; FSC, Faroe-Shetland Channel.

### Microbial relationships.

**FIG 4:**
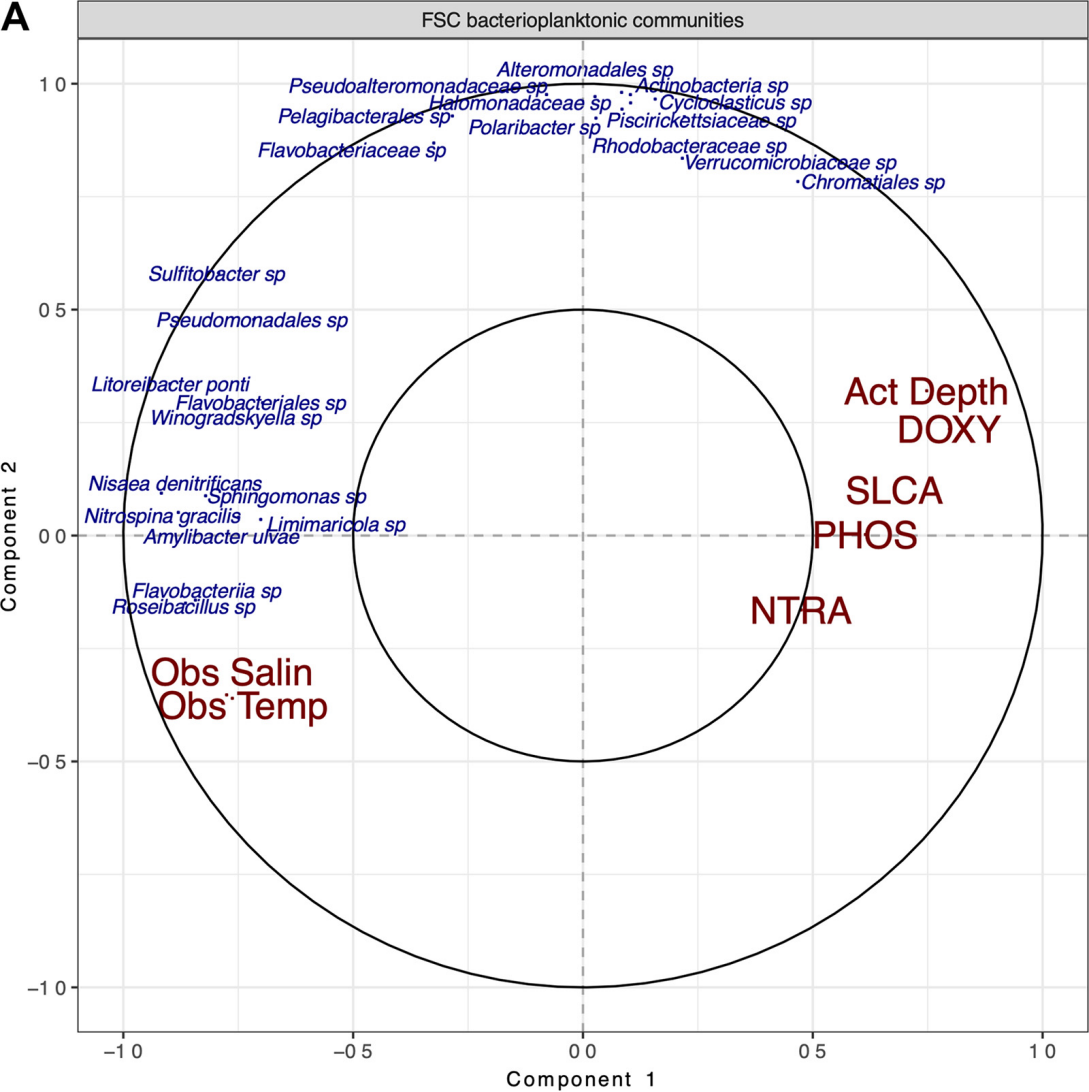

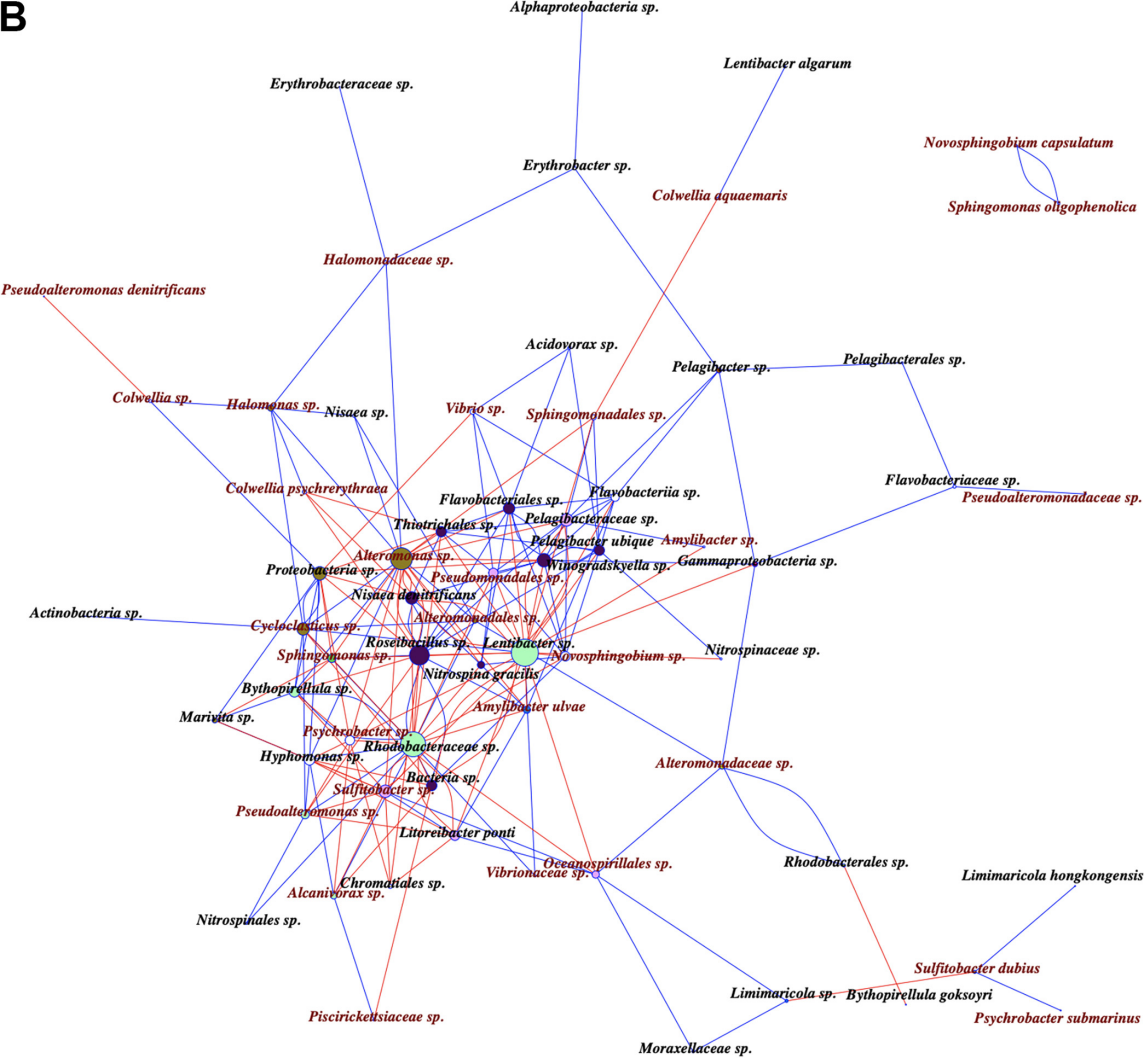
Correlations within and between phylotypes and physicochemical characteristics of the FSC. (A) Circle correlation plot based on sPLS analysis, describing relationships between phylotypes (blue) and environmental factors (red), presented in terms of radial distances and cosine angles. Sharp and obtuse angles between variables represent positive and negative correlations, respectively. The strength of correlation is represented by distance from the radial origin. Abbreviations: NTRA, nitrates (μmol/liter); PHOS, phosphorous (μmol/liter); SLCA, silica (μmol/liter); DOXY, dioxygen (μl/liter). (B) Network of significant (*P* < 0.05) cooccurrence relationships (blue edges, positive; red edges, negative) between FSC phylotypes (rho > 0.75), based on WGCNA. Known hydrocarbon degraders are highlighted in red font. Node size represents the degree of connectivity to other nodes. Node color represents the subnetworks (modules) to which the phylotypes organize.

As expected from the known hydrography of the FSC and as shown in the circle correlation plot (Fig. 4A), increased salinity and temperature were strongly and inversely related to variations in depth, oxygen, and nutrient levels. Correlations between phylotypes and physicochemical characteristics of the FSC water showed positive correlation between depth and gammaproteobacterial phylotypes, such as members of the orders *Chromatiales*, *Alteromonadales*, and *Pseudoalteromonadales*, the family *Piscirickettsiaceae*, and the genus *Cycloclasticus*. Positive correlations to salinity and temperature were observed for specific *Alphaproteobacteria* and *Flavobacteria* phylotypes, such as *Roseibacillus* spp., Amylibacter ulvae, Nisaea denitrificans, *Sphingomonas* spp., and Litoreibacter ponti. Generally, *Alphaproteobacteria* showed a slightly higher preference for oligotrophic warmer waters with higher salinity.

The network analysis (Fig. 4B) revealed significant correlations between many known hydrocarbonoclastic bacteria, observed within the FSC as well as between them and other phylotypes. The FSC network appeared to be composed primarily of three to four major, tightly intertwined subnetworks. *Gamma-* and *Alphaproteobacteria* appeared to have their own subnetworks, which are mostly negatively related to each other. Major hubs of connection within the entire network were primarily *Alphaproteobacteria* subgroups, such as *Lentibacter*, *Roseibacillus*, and other members of the *Rhodobacteraceae*. Of the *Gammaproteobacteria*, only *Alteromonas*, a taxonomic group that has also been recognized as a hydrocarbon degrader (6, 9), was recognized as a major hub of connectivity within the FSC network. This *Alteromonas* member within the FSC network was shown to have few positive associations, mostly with members of its own module, and numerous negative associations with members outside its subnetwork. This behavior suggests *Alteromonas* is a major competitor and community influencer within the FSC microbial network.

## DISCUSSION

This study explored the dynamics of the bacterioplankton communities within hydrographically distinct water masses of the FSC over two consecutive years through Illumina 16S rRNA gene sequencing. The findings revealed that the correlation between the microbial communities was more significant between water masses of contrasting physicochemical properties (water derived from different origins; e.g., NAW and NSAIW) rather than between water masses distinguished by subtle physiochemical differences (e.g., NAW and MNAW) (Fig. 2). This differentiation was also concurrent with the depth and temperature stratification of the water column, as these were properties of the water masses (Table 1). These observations corroborate those of Sunagawa et al. (23) and Lu et al. (29), who concluded that water temperature is the major driving force for community differentiation rather than any other physicochemical factor alone (e.g., depth or geography). As subtle changes of temperature, salinity, and depth did not show a significant effect on community composition (e.g., differences between NAW and MNAW communities), this study provided further insight into the thresholds of resilience of microbial communities to such factors. The differences observed in the community structure were not observed based on water mass distinguishability, which seemingly contradicted the water mass-based distinction described in the study of Agogué et al. (25), after which the current study was designed. Both ATL water masses of the FSC, however, could have originated from the same Atlantic Ocean water mass, and neither the stratification of these water parcels through the FSC, their distinct paths, or direction through the FSC nor modification from external sources was sufficient to drastically alter their microbial composition (Table 1 and Fig. 3). The same appeared true for the NOR, where mixing of the NSAIW mass with exogenous water (most likely Arctic water) altered the salinity of this water current but did not appear to affect the inherent microbial communities.

Subtle changes in temperature, salinity, depth, or water mixing did not produce differences in microbial composition; however, seasonal variation was observed within the FSC and especially the ATL water. The communities affiliated with the NOR also showed some seasonal variation, but this appeared to be due primarily to changes within the less-abundant microorganisms residing in the NOR (asserted by the weighted versus unweighted UniFrac analyses). Therefore, this variation was interpreted as potentially being a residual state from the water’s origin or history (seasonality within the Norwegian Sea or seasonal mixing with Arctic and other Nordic Seas water) rather than reflecting true seasonality occurring within the NOR. This is because the physical properties and composition of the FSC deep waters (NOR origin) have not been observed or previously reported to undergo seasonal changes (17), while seasonal microbial community, temperature, and nutrient gradients have been reported for the Norwegian Sea (27, 32–37). Thus, it is possible that the observed seasonal variability of NOR microbial communities is just a reflection of seasonal or nutritional changes within the Norwegian Sea.

Generally, the most abundant members of the FSC bacterial communities followed trends reported by global ocean baseline taxonomic microbial studies (23, 25, 27, 28). We found that *Alphaproteobacteria*, and especially the SAR11 clade (represented by *P. ubique* at >97% of total SAR11 sequences), dominated surface waters of the FSC (Fig. 3), whereas *Gammaproteobacteria* dominated the bacterioplankton communities in deeper water layers. This general community structuring corroborates those found in other deepwater and pelagic ocean environments (23, 25, 28), including elevated levels of *Actinobacteria*, *Euarchaeota*, and *Verrucomicrobia* in the deeper, colder layers of the FSC. Notably, the alphaproteobacterial genus *Lentibacter* was markedly elevated in all water masses of the FSC (∼11% abundance; Fig. 3), whereas it was observed in much lower abundance in other deepwater oceanic regions (23, 25, 28). This is quite interesting, since, previously, *Lentibacter* was not reported as a dominant, let alone ubiquitous, organism in the marine environment, and no studies report isolating a member of this genus in the FSC or other deepwater marine environment. The high abundance of *Lentibacter* in the FSC, particularly the deepwater NOR, suggests that it plays an active, yet undefined, role in the system.

We used differential abundance analysis (DeSeq2; Table 2) to reveal taxon-specific characteristics for specific water sources. This appeared especially important for the NOR, as in this water source the less abundant fraction of organisms showed the most variation and sensitivity to environmental factors (e.g., water properties and seasonal temperature variations). The background levels of some *Pelagibacteraceae* species, for example, were relatively stable across seasonal changes within the FSC (Fig. 3) and could be used to assess anthropogenic impact, resilience, and recovery of the FSC ecosystem, as in the event of a perturbation from an oil spill. The clade SAR11 is a known ubiquitous, mesophilic, pelagic member of oceanic microbial communities (38–40) and is also predictably found in the FSC, with a preference for the warmer seasons and the warmer water mass origin. Due to their sensitivity to perturbations, such as oil spills (41, 42), their abundance within the FSC could be used as indicators of oil input and subsequent ecosystem recovery. Similarly, with more knowledge and understanding of the preference of different taxa for specific water sources, including their variability across seasons and functionality within these water bodies, other phylotypes could be used as signatures of environmental or anthropogenic perturbation and for monitoring ecosystem health.

The presence and abundance of hydrocarbon-degrading taxa in the FSC (Table 3) was also assessed in this study to identify natural “seed” populations, which are capable of blooming rapidly and overtaking the background natural community in the event of a spill in this subarctic region (3, 5, 6, 11, 43, 44). The NOR contained predominantly higher abundances of hydrocarbon-degrading bacteria than the warmer, saltier ATL, especially during the spring. The higher presence of these organisms in the NOR (and fall season in the ATL), although indicative of psychrophilic behavior or possible seasonal nutrient availability, does not directly translate into a concomitant increase in biodegradative capacity, but it does reflect a robust metabolic potential for hydrocarbon bioremediation. A number of studies have documented the inhibitory effects of low temperature, oxygen, and/or nutrient levels, not only upon the activities of these organisms but also on the behavior and biodegradation rates of crude oil and its constituent hydrocarbons (45–47).

The relatively high abundance of hydrocarbonoclastic bacteria (on average, ∼15%) identified across distinct hydrographical layers of the FSC causes us to question what sustains these organisms at levels well beyond the background reported in open ocean regions. Possible explanations for these sustained high levels appear less likely to be natural (e.g., high algal hydrocarbon production for the region) due to the depth, temperature, and light availability, especially in the NOR water (Table 1). A more likely explanation appears to be of anthropogenic origin, such as in the form of small discharges of oil from oil and gas industry activity. The FSC has for many years, and continues to be, an oil-producing region that has a high density of oil drilling platforms. Hence, small discharges from industry activity could be a continual source of petroleum into this region. The heightened population of hydrocarbon-degrading bacteria in the FSC may be sustained by a continual or intermittent release of hydrocarbons from produced waters released from oil platforms directly into the sea and by frequent shipping and oil transportation activities in the region, including from the Sullom Voe, a major oil terminal on the west coast of Shetland. Although no confirmed oil seeps are known along the seabed of the FSC or in adjacent water bodies, such as the North Sea, evidence from satellite surveys suggests the presence of subsurface oil seeps on the east and west of Scotland and offshore in the North Sea (Peter Browning-Stamp, personal communication). Such persistent surface oil slicks suggest naturally occurring oil seepage, which may contribute to the elevated (above background) levels of an oil-degrading bacterioplankton population in the FSC; a similar situation is well documented in the Gulf of Mexico (11).

Recently, a few laboratory-based studies reported the microbial response of the FSC baseline communities to crude oil under representative temperature conditions (48–50). Although a number of hydrocarbonoclastic species were enriched under such conditions (e.g., *Halomonas*, *Pseudoalteromonas*, *Pseudomonas*, *Alteromonas*, *Psychrobacter*, *Oleispira*, *Cobetia*, *Vibrio*, and others), these studies included FSC seawater or sediment amendments with nutrients, oxygen, and even dispersants to compensate for the microcosm-based limitations and aid a timely microbial response. These amendments, although necessary in laboratory-based studies to observe a response, do not always accurately portray the nutrient-limiting conditions that are often a major limiting factor *in situ* during oil spills (9, 30, 49, 51). The combination of limiting nutrients and oxygen, low-temperature complex, and dynamic circulatory patterns occurring in the FSC may produce an unparalleled and strenuous challenge in the event of an oil spill not only to oil spill mitigation teams but also to the impacted ecosystem.

Although water mass-based compartmentalization did not appear to drive the microbiology of this area, this approach could be quite useful in the case of accidental oil spillage for the region. This is of particular relevance to the FSC, as this region has a significant history of oil exploration and production (1, 2), with exploration forecast to expand into its deeper waters. The identification of a hydrocarbon-degrading population (on average, 15% of the total microbial community) across seasons suggests that a future oil spill in the FSC could trigger a microbial response that differs depending on the season in which it occurs and the water mass the oil impacts or become entrained. The formation of an oil plume in the FSC, reminiscent of that which formed in the Gulf of Mexico during the Deepwater Horizon spill (52), may have more than one trajectory in the water column, depending on the water mass (depth) in which the plume forms, and any influence effected by the prevailing current(s) at the time. Therefore, oceanographic studies that involve flow predictions and current modeling (53, 54) could be crucial to understanding the fate of oil spills in the FSC and its adjacent waterways.

Of notable interest, the seasonal variability of the communities within the ATL (Fig. 3) suggests that in the event of an oil spill in the FSC, the microbial response could be quite different in the spring from that in the fall, and the same could apply during the winter and summer months. During the spring, the relative abundance of the hydrocarbon-degrading population in the ATL (∼5% of the total community) was elevated compared to that in the fall (∼4.3%) (Table 3). The higher abundance of these organisms in the spring assumes that, with respect to eliciting a microbial response, the ATL is better primed to respond to an oil spill during the spring than in the fall. As lower temperatures can inhibit hydrocarbon biodegradation (55–59), during the spring the FSC may be expected to experience a more immediate, and potentially also more effective, microbial response to an oil spill as long as conditions *in situ* are conducive to supporting the growth and activities of these types of organisms. Further work will be needed to understand the microbial response of these communities to oil and, in particular, how they might be affected by the prevailing conditions of temperature and trace and macronutrients across seasons.

This study is the first to explore the compositional variability of microbial communities in the FSC within individual water masses specific for the region and over a range of contrasting seasons and depths over a period of two consecutive years. The vertical stratification of the FSC is driven by the large contrast in water masses from two broader source regions, which provide the primary distinction underlining the difference in the microbial communities. As the geochemistry of the FSC water column experiences annual and decadal cycles (17), a reproduction of this study over a 5-year timeline, and using a more frequent monitoring strategy, could complement our reported findings and elucidate potential interannual microbial patterns. Indeed, interannual differences in community composition were detected, suggesting that a longer-term microbial monitoring is needed to fully capture and understand the microbial dynamics of the region. This could also help address questions that aim to better understand the dynamic of the hydrocarbonoclastic bacterial population in the NOR and if the high observed presence of *Lentibacter* is an annual ephemeral occurrence. As members of the *Lentibacter* genus were found elevated in this region, efforts to isolate and culture representatives of these organisms from the FSC could be included in future studies to determine their role, such as in hydrocarbon degradation, in this region.

## MATERIALS AND METHODS

### Field sampling.

Sampling of the different water masses (Table 1 and Fig. 1) was performed during the spring (24 April to 9 May) and fall (23 September to 8 October) in two consecutive years (2014 and 2015). From a long-term sample monitoring data set for the FSC (12, 13) and under the advice and guidance of experienced oceanographers from Marine Scotland Science (MSS; Bee Berx and George Slesser, personal communication), suitable locations and depths targeting the water masses NAW, MNAW, NSAIW, and NSDW were selected for sampling. Due to its inconsistent and fluctuating nature, the MEIW water mass was not targeted during this study. Sample collection was performed based on the sampling procedures of MRV *Scotia* (17). Targeted water characteristics specific for each water mass, along with the names of the most suitable stations, their geographic coordinates, and depth of collection, are presented in Table 1. Samples from the distinct water masses were collected using 10-liter Niskin water bottles mounted on a CTD (conductivity, temperature, depth) carousel. The CTD traces are shown in Fig. S1 in the supplemental material. Three replicates of 1-liter volumes from each targeted water mass were immediately filtered through individual sterile 0.22-μm-pore-size (47-mm-diameter) gridded mixed cellulose ester filters (Millipore Sigma). Filters were stored at −20°C for subsequent processing. Salinity and temperature characteristics at depth were acquired for each sample collected.

10.1128/mBio.03701-20.1 FIG S1 Water column CTD profile for sampling station NOL08 (A), station FIM03 (B), station NOL07 (C), and station FIM6a (D). The arrow indicates sampling depth at each station. Download FIG S1, DOCX file, 0.2 MB. Copyright © 2021 Angelova et al. 2021 Angelova et al. https://creativecommons.org/licenses/by/4.0/ This content is distributed under the terms of the Creative Commons Attribution 4.0 International license.

### DNA extraction, amplification, and Illumina MiSeq sequencing.

For DNA extractions, one-third of each filter was crushed into liquid nitrogen to fine powder. The subsequent genomic DNA extraction workflow was based on the protocol of Tillett and Neilan (60). Along with every set of marine samples processed, a set of negative controls (blank filters) was included to take into account any exogenous DNA introduced during the extraction and subsequent processing. Extracted DNA was confirmed using a NanoDrop 3300 fluorescence spectrometer (ThermoScientific) and further confirmed by gel electrophoresis. PCR amplifications of the V3-V4 16S rRNA gene fragment were performed on all genomic DNA using MyTaq polymerase (BioLine), as described by the manufacturer’s protocol. Primer sequences of choice were the universal bacterial primers of forward or reverse direction, 341F (CCTACGGGNGGCWGCAG) and 785R (GGACTACHVGGGTATCTAATCC) (61). Target-specific primer pair coverage distribution, tested with the TestPrime 1.0 tool against the SILVA RefNR database (62), included 85.9% bacterial, 0.6% archaeal, and 0% eukaryotic coverage. Negative controls (molecular-grade water) were employed to check for DNA contamination during the PCR procedure. PCR products were quantified by NanoDrop (as described above) and checked by gel electrophoresis. PCR products (∼440 bp in size) were enzymatically cleaned of polymerase and residual primers using 10 U exonuclease I and 1 U FastAP (ThermoFisher) according to the manufacturer’s protocol (ThermoFisher).

Illumina MiSeq sequencing was performed at the University of Liverpool, Centre for Genomic Research. For this, two out of the three replicates from each sample were selected. At the sequencing facility, the PCR products underwent a second round of PCR (8 cycles), adding barcodes and the Illumina Nextera XT adapters of the amplified sequences per the adapted protocol of Berry et al. (63). This two-round PCR approach has been shown to reduce PCR bias and sequencing artifacts that are especially common for complex environmental multibacterial samples (63). Prepared sequencing libraries were qualified and quantified using Qubit Fluorometer and Bioanalyzer systems (ThermoFisher Scientific) and run on a 2 × 250 paired-end run of the Illumina MiSeq platform. Demultiplexed and primer-trimmed data files were returned for downstream processing, as described below.

### Sequence data preprocessing.

Sequenced replicates were treated separately throughout the following bioinformatics pipeline, and counts were averaged after clustering. Forward and reverse sequences with an overlap of >10 bp were merged using PANDAseq assembler (v 2.7 [64]) and then qualified using PRINSEQ software (65). PRINSEQ-lite (v 0.20.4 [65]) software was also used to trim ends of reads with quality scores of ≤35 and filter out reads with total quality scores of <30 and/or lengths below the mean length of the sample (∼15 to 20% of reads). Thus, good-quality reads used for downstream analysis were 390 to 460 bp in length and had a quality score of >30. The sequence analysis tool VSEARCH (version 2.15.1 [66]) was used to perform 16S rRNA gene denoising, chimera checking, ASV selection, and taxonomic assignment from the quality sequence data. The analysis pipeline involved dereplication of the reads, denoising, and clustering of reads to generate amplicon sequence variants (ASVs; UNOISE3 algorithm), screening of chimeric and homopolymeric sequences, and taxonomic assignment of the selected ASVs (SINTAX algorithm). VSEARCH was also used to create the ASV absolute abundance (counts) table according to the user manual and default settings. Taxonomy was assigned to representative sequences (ASVs) to the deepest assignable taxonomic rank (minimum 75% confidence level threshold). The databases used for taxonomic assignment included UTAX-formatted versions of the 16S rRNA gene microbial BLAST database (67) and SILVA 16S rRNA gene database (62). Due to the uncultured nature of many microbes, not all ASVs could be assigned a species-level taxonomy. In those cases, the deepest possible taxonomic assignment was used. ASVs are also referred to in this text as phylotypes.

### Statistical, biological, and community analysis.

Community profiles, biological indices, and biostatistical discriminant analyses (sparse partial least squares [sPLS-DA] and DESeq2) and network analysis (WGCNA) were performed using the R language (v 3.3.0) in combination with RStudio software assembly (68, 69) and the latest available versions of various bioinformatics packages (ampvis2, phyloseq, vegan, GUniFraq, DESeq2, microbiomeSeq, sinkr, and mixOmics [70–76]). sPLS-DA was performed using R package mixOmics and visualized via a circle correlation plot (77). Network analysis at species level was performed based on WGCNA algorithms (code courtesy of Umer Ijaz, University of Glasgow).

To exclude ASVs with low biological reliability (assumed likely PCR or lingering sequencing artifacts), ASVs were additionally filtered to a prevalence of 20% and cumulative abundance of 15% prior to relative abundance normalization and downstream analysis. Alpha diversity indices were calculated on the filtered and normalized ASV table. Alpha diversity variance analysis (ANOVA) was performed on all indices between designated groups (water mass origin, water mass, and season) to assess the significance of community variance within and between the groups. Beta diversity was assessed via permutational multivariate ANOVA (PERMANOVA) and analysis of similarity tests (ANOSIM), based on the Bray-Curtis dissimilarity matrix.

Taxonomic relatedness of communities based on origin of the water mass and seasons was assessed and visualized via UniFrac analyses (qualitative/weighted and quantitative/unweighted; GUniFrac and pyloseq R packages) within FSC distinguished water masses. PERMANOVA tests were also performed on the UniFrac-based matrix to assert the significance of cluster separation in the UniFraq analyses. The significance of all statistical tests was based on a *P* value cutoff of 0.05 (under 5%). ASVs showing a significant change (*P < *0.05) in relative abundance between water mass origin and across seasons were revealed via DESeq2 algorithms and classified using a random forest classifier implemented by the R package microbiomeSeq (version 0.1) (77). For the construction of community profile heatmaps, samples were grouped and ASV abundance averaged based on water masses, origin of those water masses, and seasons. Furthermore, the FSC community profiles were examined to identify sequences matching known and putative hydrocarbon-degrading bacteria reported in the literature. The average abundance of each phylotype (with abundance of >0.01%) within each water mass origin community profile and season was determined.

### Data availability.

All sequences were deposited in the SRA repository under no. PRJNA682843, and all scripts, code, and additional materials related to this project can be found at https://github.com/angelovaag/FSC_scripts.git.
