# Supplementary material for: Inter- and Intra-Annual Bacterioplankton Community Patterns in a Deepwater Sub-Arctic Region: Persistent High Background Abundance of Putative Oil Degraders
Source: mBio. 2021 Mar 16;12(2):e03701-20. doi: 10.1128/mBio.03701-20 (PMC8092327; doi:10.1128/mBio.03701-20)

**Supplementary Data**

**Supplementary Figure 1.** Water column CTD profile for sampling station NOL08 (A), station FIM03 (B), station NOL07 (C), and station FIM6a (D). Arrow indicates sampling depth at each station.


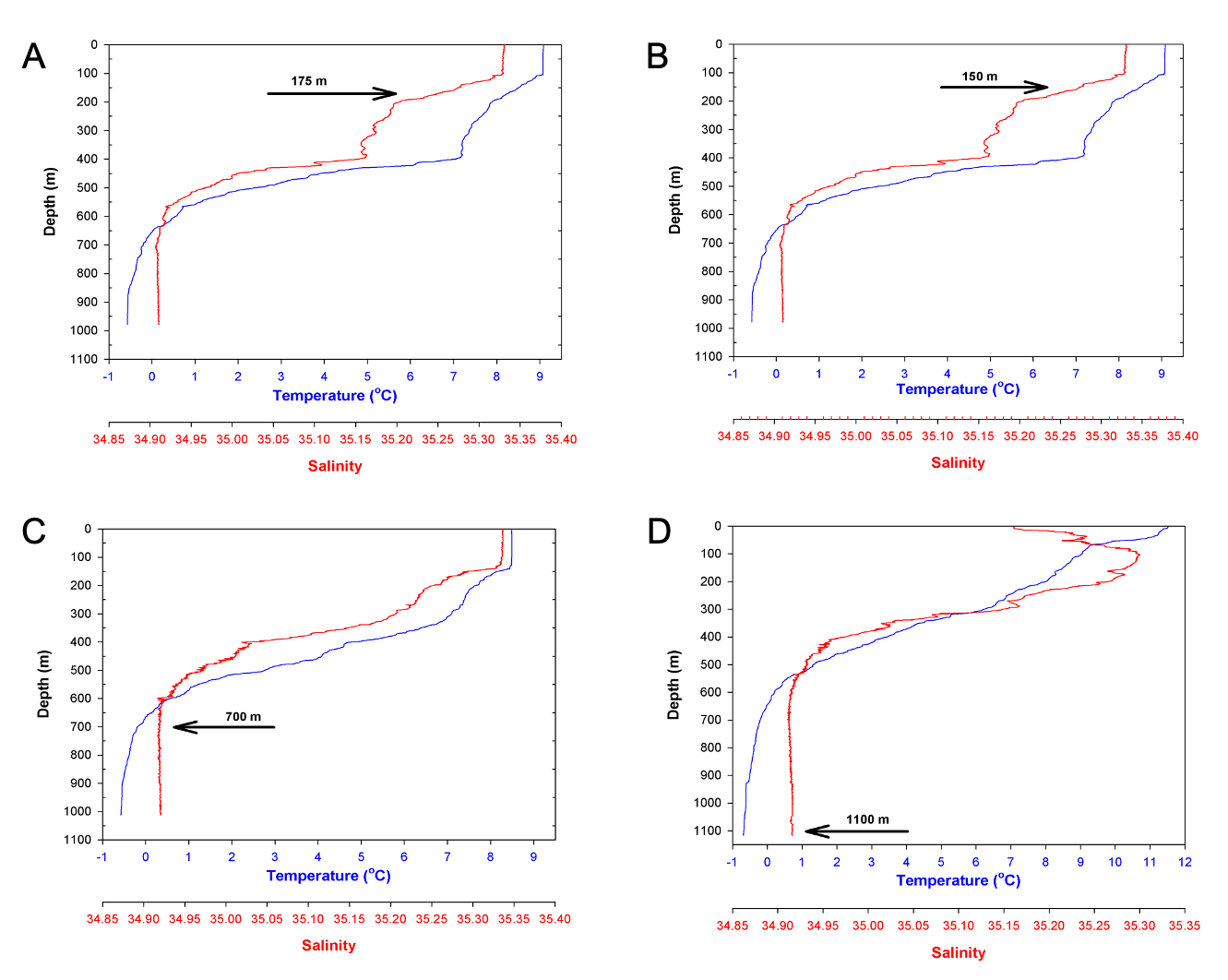

Supplement: FIG S1 [file mBio.03701-20-sf001.docx]
